# Fast Undersampled Functional Magnetic Resonance Imaging Using Nonlinear Regularized Parallel Image Reconstruction

**DOI:** 10.1371/journal.pone.0028822

**Published:** 2011-12-14

**Authors:** Thimo Hugger, Benjamin Zahneisen, Pierre LeVan, Kuan Jin Lee, Hsu-Lei Lee, Maxim Zaitsev, Jürgen Hennig

**Affiliations:** Medical Physics, Department of Radiology, University Medical Center Freiburg, Freiburg, Germany; University of Nottingham, United Kingdom

## Abstract

In this article we aim at improving the performance of whole brain functional imaging at very high temporal resolution (100 ms or less). This is achieved by utilizing a nonlinear regularized parallel image reconstruction scheme, where the penalty term of the cost function is set to the L_1_-norm measured in some transform domain. This type of image reconstruction has gained much attention recently due to its application in compressed sensing and has proven to yield superior spatial resolution and image quality over e.g. Tikhonov regularized image reconstruction. We demonstrate that by using nonlinear regularization it is possible to more accurately localize brain activation from highly undersampled k-space data at the expense of an increase in computation time.

## Introduction

Conventional functional magnetic resonance imaging (fMRI) is performed using multi-slice EPI with TR of 2–3 s. Recently a number of new methods [1], [2] have been suggested to speed up data acquisition well below 1 s. One incentive for doing so is the ability to remove physiological noise from the time series data, which in combination with the increased number of sampling points per unit time increases the sensitivity of the fMRI acquisition dramatically. In the extreme case, Magnetic Resonance Encephalography (MREG) or Inverse Imaging (InI), have been suggested, which allow extremely fast acquisition by omitting gradient encoding altogether and acquiring spatial information from the small sensitive volumes of multi-array coils alone. By adding some gradient encoding using highly undersampled acquisition with multi-coil image reconstruction, full-brain datasets with acquisition times of 100 ms can be acquired [3], [4].

The strongly undersampled trajectories used in these studies lead to very high undersampling factors and prohibit a conventional non-cartesian image reconstruction using e.g. SENSE (sensitivity encoding) [5], [6] or other parallel imaging methods. Tikhonov regularization [7], [8] was therefore previously employed to find a sensible solution to the ill-conditioned reconstruction problem. Another reconstruction approach for highly undersampled data was taken by Lin et al., where they used reconstruction techniques usually found in radar and magnetoencephalography literature [9]. Furthermore, they also utilized a GRAPPA-based k-space reconstruction method [10]. Recently, Lee et al. have shown that by using interleaved data acquisition, a single channel coil, density compensated non-uniform Fourier transformation and UNFOLD [11], [12], the temporal resolution of an fMRI experiment can also be increased. Their approach relies on temporal filtering of the reconstructed data, which can potentially affect physiological signal components (BOLD or otherwise). Rabrait et al. have employed cartesian Echo Volumar Imaging (EVI) to achieve repetition times for functional imaging of up to 200 ms [13]. They employ Tikhonov regularization to stabilize the inverse problem. Nonlinear regularization techniques have also gained strong attention over the last few years in MRI, since they have the potential to yield better image quality when compared to linearly regularized approaches with an equal amount of k-space data [14], [15].

In this work, we try to push the spatial resolution of fast, undersampled functional imaging at 10 Hz or more by utilizing nonlinear regularized reconstruction methods and non-cartesian k-space data sampling to improve the point spread function compared to standard cartesian sampling.

## Methods

### Ethics statement

All experiments on human subjects were performed with approval by the ethics committee of the Albert-Ludwigs university of Freiburg, Germany and all subjects gave written informed consent before commencement of the study.

All experiments were performed on a 3T Magnetom Trio (Siemens, Erlangen, Germany). For signal reception a commercial 32-channel head array was used.

### fMRI paradigms

A checkerboard paradigm was used for stimulation of the visual system, with an inversion frequency of 4 Hz. It was presented in a block design with 3 periods that consist of 15 s of activation followed by 15 s of rest.

The subject also had to fixate a dot in the middle of the checkerboard during the whole experiment. After 12 s, the dot changed color for 5 s. During that time the subject was requested to perform bilateral finger tapping. For each data set, the initial rest period of 15 s was removed from the data set prior to image reconstruction to avoid the initial signal transition into the steady state.

### Trajectory

Since an acquisition scheme with a very high undersampling factor is used here, the choice of the trajectory plays a crucial role in the ability to accurately localize the activation. Standard Cartesian undersampling is a poor choice, because it leads to aliasing artefacts in the reconstructed image. For high undersampling factors *R*, the aliasing cannot be completely removed and the point spread function (PSF) will have characteristics of a comb function (when neglecting the finite sampling) with *R* peaks within the field of view. As a result, aliasing and therefore BOLD signal changes will occur all across the image and localizing the activation becomes very difficult.

Ideally, a more appropriate trajectory for this task is radially symmetric, since symmetry in the sampling pattern is carried over to the shape of the corresponding PSF. The expected PSF of such a trajectory is much more benign. Here we chose a 3D single shot radial sampling (SSR) strategy, with a very low number of spokes per T_R_. Due to the very low number of spokes, the radial symmetry of this trajectory is strongly reduced and the PSF of such a trajectory exhibits side lobes that lead to the typical streaking artefacts of radial sampling, but at a lower distance from the PSF center compared to full radial sampling.

**Figure 1:**
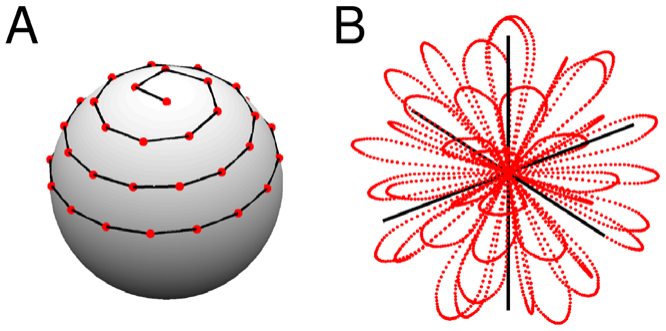
Design of the trajectory. End points of the radial sections of the trajectory (A) and 3D plot of the k-space sampling pattern (B).

The actual trajectory with N_P_ = 40 zero crossings can be seen in fig. 1b. N_P_ = 40 yields an acquisition time of 32 ms which was chosen as a balance between the number of k-space points and severity of off-resonance effects during signal acquisition. The radial k-space lines where arranged according to a homogeneous sampling pattern on a sphere. First, following [16], N_P_ points were defined (see fig. 1A) on a hemisphere. These points define the orientation of the radial spokes. Two neighbouring spokes were connected using an optimization approach which finds the shortest gradient wave form within the hardware limits that connects the two end points of the spokes [17].

### Data acquisition

The following measurements comprise a complete session for one subject:

1. A reference measurement was performed, which consisted of a multi-slice acquisition of a slab that covered the whole brain of the subject. The field of view in both directions was set to 256 mm with a resolution of 64×64, and 64 slices were recorded with a slice thickness of 4 mm, yielding isotropic voxel size. Two echos per T_R_ were acquired, which enables the determination of a map of the off-resonances due to field inhomogeneities. The parameters of the sequence were T_R_ = 25 ms, T_E1_ = 4.9 ms, T_E2_ = 7.4 ms and a flip angle of 15°. The data was saved separately for each coil. This reference data was used to synthesize the coil sensitivities by dividing each coil image by the adaptive coil combination [18]. An off-resonance map was computed by determination of the voxel phase evolution between the images of the two echo times. The resulting map was additionally smoothed using a Gaussian filter with a width of 1 voxel to reduce noise in regions of low signal intensity.
2. For comparison of our methods, the checkerboard stimulation was performed using multi-slice T_2_*-weighted EPI as imaging sequence. 24 slices with an isotropic voxel resolution of 2 mm were recorded with a repetition time per slab of T_R_ = 2 s. The flip angle was set to 90° and the echo time was equal to T_E_ = 30 ms.
3. MREG-fMRI was performed using simultaneous checkerboard and finger tapping stimulation. The flip angle was set to 15°, and the gradient scheme that corresponds to the optimized 3D radial trajectory was played out. The trajectory started 5 ms after the excitation pulse and had a total duration of 32 ms. The repetition time was set to T_R_ = 100 ms.
4. Measurement of the trajectory was performed according to the thin slice method given by Duyn et al. [19].

### Image reconstruction

The standard multi-coil signal equation is cast into a linear system of equations:
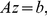
(1)here 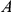 is the encoding matrix, 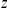 is the unknown image and 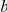 is the acquired data of all coils of a single time frame. To clarify the structure of 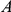, it can be decomposed into the following form:
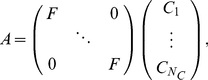
(2)where 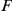 is the non-uniform Fourier transformation (in the actual implementation the fast non-uniform Fourier transformation (nuFFT) [20] is used), and 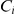 is a diagonal matrix, with the coil sensitivity of the coil *i* on its diagonal. The task of computing the unknown image 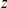 from the measured data 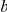 becomes an under-determined inverse problem. Standard imaging reconstruction using the pseudo-inverse fails here, since the encoding matrix 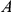 in the forward equation is usually strongly ill-conditioned due to strong undersampling and the non-optimized coil sensitivity coverage. The problem therefore needs to be regularized to find a sensible solution. A relatively simple regularization method is the well known Tikhonov-regularization [7], [8]. In this case image reconstruction is defined as the minimum of the cost function:
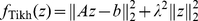
(3)where 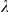 is the regularization parameter. Since the solution to this optimization problem is a compromise between the two terms in the cost function, depending on the choice of the regularization parameter, a certain deviation from an exact solution of equation (1) has to be accepted at the expense of a 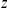 that has a smaller L_2_-norm. This generally leads to a preference for smooth images.

Recently the L_1_-norm as a penalty in the cost function gained popularity since it has more desirable properties with respect to image reconstruction from undersampled data than the conventional Tikhonov penalty. As a regularization penalty, it is known to have edge-preserving properties. Furthermore, in [21] the L_1_-norm was shown to yield solutions that have an approximately sparse representation in the domain in which it is measured. This can be exploited in the case where the unknown image is known to have a sparse representation in some transform domain. In general, a good choice for such a transform is the wavelet transform, since most images can be approximated with high precision with only a strongly reduced number of their wavelet coefficients. Thus the following cost function is used for image reconstruction:
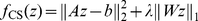
(4)where 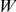 represents a transform and 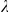 is the regularization parameter.

While regularization methods offer the possibility of reconstructing images in ill-conditioned situations, the main obstacle of using such a method lies in the fact, that the degree of regularization that is introduced in the reconstruction must be specified and there is usually no universal rule to pick the right amount. For example, in truncated singular value decomposition, regularization is controlled by the number of singular values that are retained. Another example is the conjugate gradient method, which is known to yield a regularized solution if it is stopped before convergence. Thus, the amount of regularization is controlled by the number of iterations. In approaches based on cost functions, like ours, the degree of regularization is determined by the regularization parameter.

Several methods to automatically determine the regularization parameter exist for Tikhonov regularization. Here the L-curve method [22] was used. The successful application of this method to Tikhonov regularized parallel imaging was demonstrated in [23]. In the nonlinear case 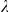 was chosen empirically, i.e. the time series was reconstructed for different values of 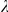 and the most reasonable value compared to the EPI activation maps was chosen. This parameter was reused for subsequent reconstructions. A nonlinear conjugate gradient [24] procedure was used to find the minimizing solution of 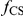, while the Tikhonov cost function was minimized using the linear conjugate gradient method [25]. No additional post-processing steps were performed after image reconstruction of an image time series.

### Statistical analysis

General linear model analysis was performed on the resulting image time series. The GLM-analysis was carried out using SPM8 (Statistical Parametric Mapping, www.fil.ion.ucl.ac.uk/spm). In all activation maps shown in this article voxels are identified as activated, when a family wise error rate of 5% for the null hypothesis (i.e. voxel is not activated) was exceeded.

### Off-resonance correction

Additionally, signal degradation during acquisition due to field inhomogeneities can be incorporated in the reconstruction process. To accomplish this, the off-resonance map 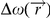 that is determined from the reference data is used to model the additional off-resonance phase factor in the signal equation:
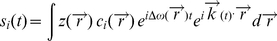
(5)


In this form, it is not possible to apply the nuFFT directly anymore, since the off-resonance phase factor depends on time and position. A time segmented approximation [26] is used to maintain the possibility of using the nuFFT in the conjugate gradient algorithm:
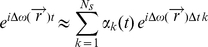
(6)


In this way, the integration in equation (5) can be performed on each summation term and the time dependence can be pulled out of each integration. With this approach, the computation time is essentially increased by a factor equal to the number of segments 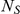. The new modified forward model 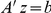 now has the following decomposed encoding operator:
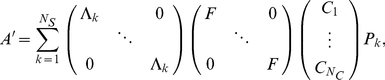
(7)where the 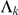 are diagonal matrices containing the weighting functions 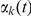 and 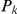 is another diagonal matrix containing the phase factor 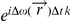.

## Results

**Figure 2:**
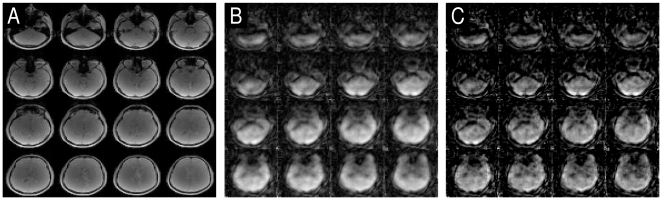
Sample reconstructions. Sum of square image of the reference data (A), a sample reconstruction using the Tikhonov regularization (B) and the sample reconstruction using the l_1_-norm regularized reconstruction (C).

Figure 2 shows a comparison between a reference image (A), a reconstructed time frame of a time series using Tikhonov regularization (B) and using L_1_-norm regularization (C). The reconstructed images have a matrix size of 64×64×64 with an isotropic resolution of 4 mm per voxel. Only the central 16 slices are plotted here. Due to the extreme undersampling, the image quality is necessarily inferior to the fully sampled case. Blurring and signal cancellations due to the radial signal acquisition and off-resonance effects are observed in both undersampled reconstructions. Images reconstructed with L_2_-norm appear smoother compared to L_1_-norm reconstruction, L_1_-norm images are sharper and of better quality. This is expected since the L_1_-norm has superior properties with respect to image reconstruction. We will show that this improved behaviour compared to the Tikhonov reconstruction also carries over to an improved spatial localization of activation in functional MRI when extreme undersampling is used to push the temporal resolution.

**Figure 3:**
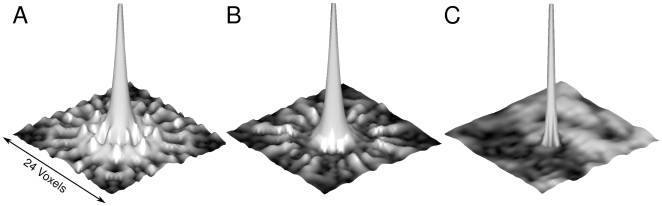
Point spread functions. A PSF for the 3D rosette trajectory (A) and for the 3D SSR (B) in the Tikhonov regularized case in comparison to a local PSF of the 3D SSR trajectory using the L_1_-norm regularized reconstruction (C).

The actual spatial resolution can be characterized by the point spread function (PSF). For a given test image 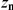 which is equal to one in the voxel of interest 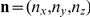 and zero everywhere else, the PSF in case of a linear reconstruction is defined as:
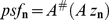
(8)where 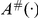 denotes the reconstruction operation. For the Tikhonov regularization 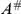 has a well known analytic form and can be expressed as a matrix. In case of a nonlinear cost function a PSF can not be readily defined, since then the operation 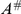 is nonlinear as well. The solution thus not only depends on the position as in Tikhonov regularization, but on the measured data as well. An image dependent PSF can still be defined by computing the difference with respect to an underlying image 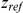 [27]:
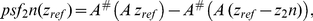
(9)where 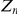 needs to be only a small perturbation to 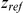. Figure 3 shows a comparison of the PSF at a position in the middle of the field of view (FOV) for two different trajectories in the Tikhonov case. Only a transversal slice through the center of the three dimensional PSF is shown. In fig. 3A the PSF for a 3D rosette trajectory is shown, which was used in a previous study for fast functional MRI [4], while in fig. 3B the PSF for the 3D SSR trajectory is shown. Judging from the PSF, the SSR is a better choice with respect to the achievable spatial resolution, due to the higher isotropy of the trajectory. The strong side lobes in the rosette PSF vanish and only smaller side lobes appear further away from the center of the PSF. In fig. 3C the PSF given by equation (9) for the L_1_-norm regularized reconstruction and the SSR trajectory is then plotted, where a reference image of a slice of the brain was used for 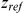. The width as well as the side lobes are greatly reduced compared to the Tikhonov reconstructed PSF, demonstrating the superior image reconstruction properties of the L_1_-norm.

**Figure 4:**
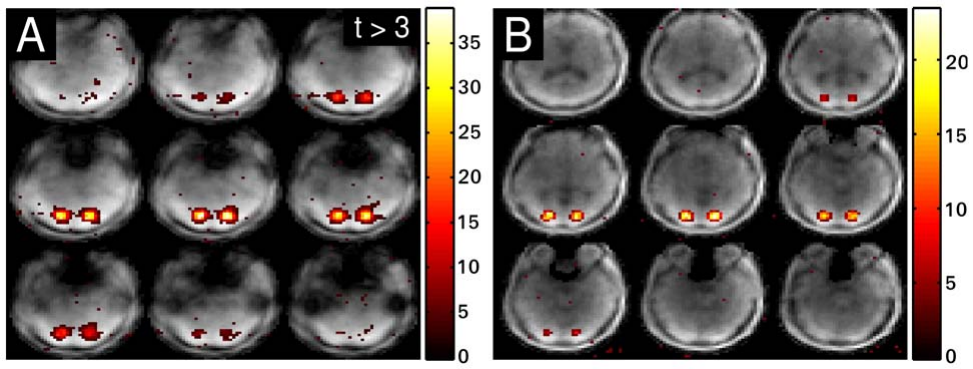
Simulation of brain activation. Results of the simulation: Tikhonov reconstructed time series (A) and L_1_-norm regularized reconstruction (B).

Spatial localization has also been assessed by numerical simulation. For simulation, a static time series data set was generated using a 64×64×64 3D reference image of the brain. In voxels belonging to two 3×3×3 cubic regions separated by 6 voxels, a simulated BOLD response was added. The corresponding k-space data set was generated by applying the forward model 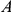 to each time frame. Noise with a relative intensity of 1% was then added to the k-space data. Each time frame was reconstructed using L_1_- and L_2_-norm penalties in the cost function. The reconstructed time series was analyzed using a GLM-analysis, and thresholded corrected t-values were overlaid on top of the first reconstructed time frame of the time series. A comparison of the results is plotted in fig. 4, where (A) corresponds to the L_2_-norm reconstruction and (B) corresponds to the L_1_-norm reconstruction with 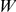 equal to identity. Both figures show 9 slices across the activated region out of the reconstructed 3D volume.

The simulation demonstrates the improved performance of the L_1_-norm reconstruction over the L_2_-norm reconstruction with respect to spatially localizing the activation in the two cubic regions of the brain. The L_2_-norm reconstruction exhibits much more pronounced blurring and spread of activation into voxels outside of the two cubic regions. Blurring is strongly reduced, when the L_1_-norm is used. The improved spatial resolution of L_1_-norm is also directly observable from the overall image appearance, with a better definition of the CSF-filled spaces after L_1_-norm reconstruction.

**Figure 5:**
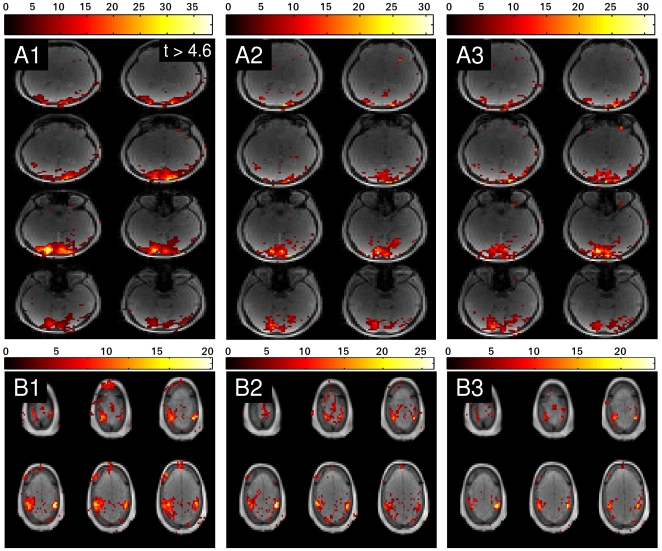
Comparison of activated brain areas for different reconstructions. Penalty terms: Tikhonov (column 1), l_1_-norm (column 2) and l_1_-norm in the wavelet domain (column 3) for visual checkerboard stimulation (row A) and bilateral finger tapping (row B).

In fig. 5 the results of the visual checkerboard stimulation (row A) and the motor task (row B) are displayed. Column 1 corresponds to the Tikhonov reconstruction, column 2 corresponds to the L_1_-norm reconstruction with 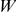 equal to identity and column 3 corresponds to the L_1_-norm reconstruction with 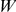 equal to a wavelet transformation. Again, only relevant slices in the complete 3D volume are displayed. The measured results agree with the observations from simulation. L_1_-norm reconstruction delivers better spatial localization of the visual and the motor activation. Using a wavelet transform in the L_1_-norm penalty does not lead to an appreciable difference. It can be observed that differences in t-scores between different reconstruction methods occur. Firstly, these can be attributed to the different properties of L_1_-norm and L_2_-norm, which will affect the voxel time courses and thus the t-scores. Secondly, the empirical choice of the regularization parameter in the L_1_-norm case will also affect the t-scores.

**Figure 6:**
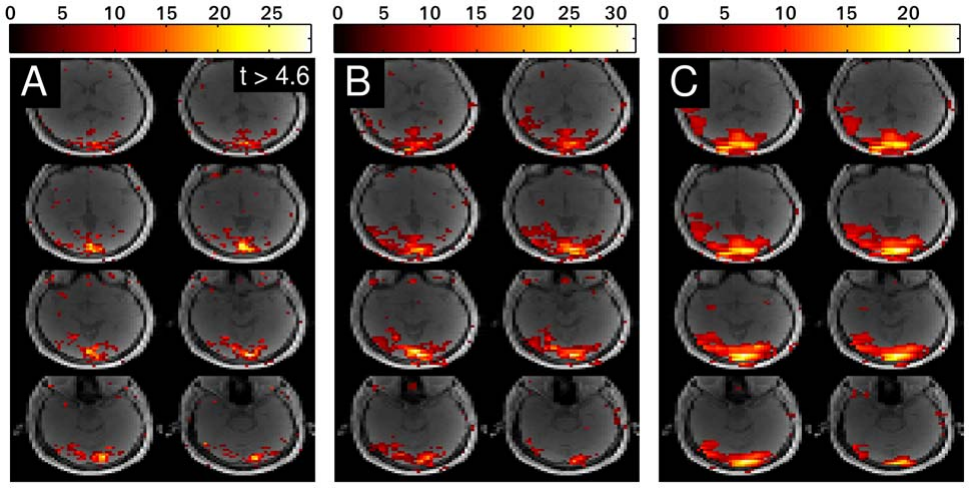
Activation map dependence on the regularization parameter. Image reconstruction was performed using the l_1_-norm penalty and the following regularization parameters: 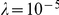 (A), 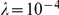 (B) and 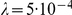
(C).

In fig. 6 activation maps are plotted for the visual checkerboard task for L_1_-norm penalized reconstruction using a wavelet transform for 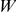. From left to right, increasing regularization parameters were employed in the cost function. The typical behaviour of over- and under-regularizing the reconstruction can be observed here. Choosing a small value for 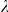 leads to low SNR-images and activation is lost in the amplified noise. For 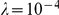, more sensible results are obtained. If 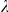 is chosen too large, the influence of the penalty term over the data fidelity term eventually becomes too great.

Non-cartesian single-shot trajectories are very sensitive to off-resonance effects from field inhomogeneities. The acquisition time for the 3D-SSR trajectory is 32 ms, during which significant magnetization dephasing due to off-resonance effects will take place. This will lead to inconsistencies especially at and around the crossing points of the trajectory. As a consequence, the signal will be degraded and image quality is expected to be affected substantially. Taking off-resonance effects into account using the modified forward model (7) in the image reconstruction procedure is therefore expected to improve image quality.

**Figure 7:**
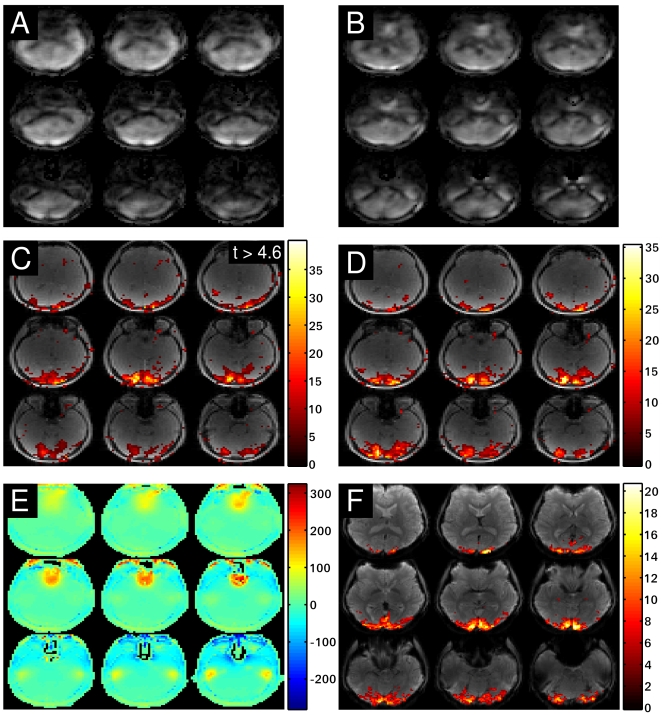
Comparison with additional off-resonance correction. Sample L_1_-norm-penalized reconstruction without (A) and with additional off-resonance correction (B).Activation map without (C) and with (D) off-resonance correction. A spatial map of the off-resonances in Hz (E) and the activation map of an EPI experiment (F).

Off-resonance correction was performed by setting the number of time segments in equation (6) to 10. The results for one subject are shown in fig. 7. The figure shows a comparison of a L_1_-norm penalized reconstruction versus one with the additional incorporation of an off-resonance map in the forward model. The comparison of uncorrected (A) versus corrected sample reconstruction (B) demonstrates that the correction is able to recover signal voids that appear in the uncorrected sample image.

The activation map derived from the off-resonance corrected time series (D) exhibits more pronounced features compared to the uncorrected version (C). The agreement with the activation map from a conventional EPI experiment (F) is greatly improved by the additional off-resonance correction. The identified brain regions are virtually identical when compared to EPI. The blurring of the activation in the t-maps is also significantly reduced. Furthermore, the activation map is more symmetric, as expected when using a simple checkerboard paradigm. The asymmetry in the uncorrected activation map reflects the asymmetric k-space attenuation due to off-resonance effects and the order in which the radial sections of the sampling scheme are acquired. It would be interesting to directly compare the activation maps using EPI and the SSR statistically, unfortunately this rather difficult due to the fact that the two data acquisition schemes lead to very different artefacts. While EPI typically leads to distortions, the SSR tends to introduce blurring.

**Figure 8:**
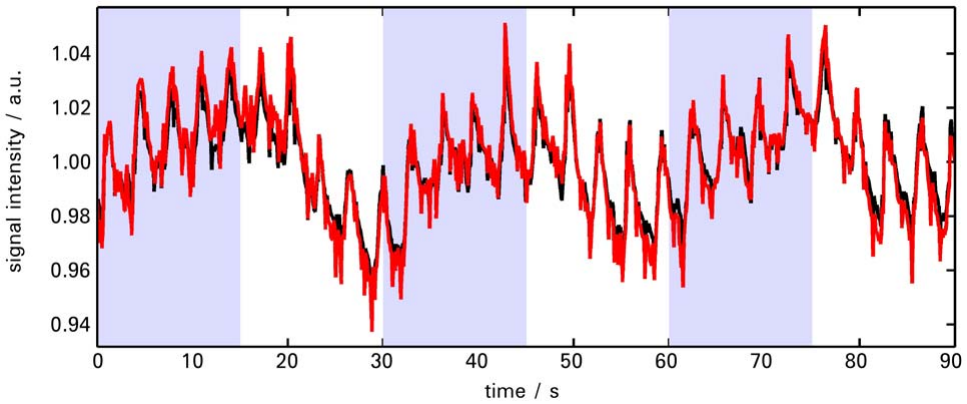
Comparison of time courses. Time course of a voxel in the visual cortex for Tikhonov (black) and l_1_-norm regularized reconstruction (red). The sections with the light blue background correspond to the times when the checkerboard was switched on.

A typical time course picked from the visual cortex for the Tikhonov (black) and the L1-norm penalized case (red) can be seen in figure 8. Time courses have been normalized by dividing by their mean. Time courses exhibit excellent agreement. Periodic modulations correspond to the breathing component.

## Discussion

Our study demonstrates the feasibility of functional MRI using highly undersampled k-space trajectories to increase the achievable sampling speed. The key to being able to still properly localize the activation even with extreme reduction factors is a suitable image reconstruction scheme in combination with suitable receive coil arrays. While simpler reconstruction methods for non-cartesian k-space data, e.g. gridding, are fast and robust in the conventional imaging regime where Nyquist-conform sampling is employed, these techniques fail to deliver usable results when moved to the strongly undersampled regime. The ill-conditioning of the parallel imaging reconstruction problem due to undersampling and the geometry of the receive array makes regularizing the solution a necessity. In previous work, Tikhonov regularization was used to deal with the inverse problem, here nonlinear regularization was able to further improve the spatial localization of the activation. It was found that a L_1_-norm penalty in the cost function is better suited to the reconstruction problem and yields better results than the simpler L_2_-norm penalty, due to the edge-preserving properties of the L_1_-norm. Furthermore, we found that an additional wavelet transform to measure the L_1_-norm in a sparser domain does not lead to an improvement in the reconstruction performance, although, for the sparsification or compressibility of a brain image, a wavelet transform is in general a superior choice to using no transformation (i.e. identity). Nevertheless, similar results were obtained with both transforms. This can be explained by the lack of a random sampling pattern, a crucial missing prerequisite for the application of compressed sensing. Only then do pseudo-random noise artefacts appear due to random sampling, and a sparse transform domain can help to remove them.

Here, we only considered the application of a single L_1_-norm penalty term in the cost function. Penalty terms that are better suited to the task of activation localization might exist and lead to further improvements. Furthermore, the combination of more than one penalty term has also been shown to be potentially beneficial to image quality [14].

One disadvantage of dealing with the non-cartesian SENSE equation is the necessary estimation of the coil sensitivities. K-space-based methods are much more flexible in this respect. Also, it was reported that a more sensitive detection of BOLD activation can be achieved using a GRAPPA-based reconstruction approach [10]. Recently, a technique abbreviated L_1_-SPIRiT [28] was introduced, that allows regularization methods to be applied to GRAPPA-based image reconstruction. This might be well-suited to our purpose, since movement related issues with stationary coil sensitivities during SENSE based reconstruction will be greatly reduced.

The results also demonstrate significant improvements by incorporating the field inhomogeneity correction. Although field inhomogeneity dependent frequency variations are rather small across the visual cortex (±10 Hz in fig. 7E), activation maps are improved.

The use of a nonlinear cost function necessitates replacement of the standard conjugate gradient with a nonlinear conjugate gradient algorithm. This leads to an increase in the computation time, since the directions of the descending steps lose their conjugacy. Even with periodic restarts, this greatly increases the number of steps it takes to reach convergence. Additionally, a time consuming line search is necessary in each step. In our experience, roughly an order of magnitude more time is necessary to reconstruct a time frame with the L_1_-norm penalty compared to the L_2_-norm penalty. With a temporal resolution of 100 ms or less and fMRI experiments that last typically for several minutes, reconstructing a complete time series is a time consuming task. Compared to only a few seconds with a standard FFT plus sum-of-squares reconstruction in the fully sampled case, reconstructing a single timeframe using the nonlinear regularized reconstruction takes about 5 min (100 NLCG-iterations). Grid computing and GPU-accelerated algorithms can tremendously alleviate this issue. Additional off-resonance correction further increases the reconstruction time essentially by the number of time segments that are used.

It has to be noted that SPM8 was used to perform the GLM analysis on each voxel of the reconstructed time series. Although SPM8 is a tool that is designed to analyze functional MRI data, it uses two underlying assumptions that are not necessarily valid anymore for our kind of data. One is the assumption that the temporal resolution of the time series is rather low. This is indirectly assumed by setting the autoregressive model (of order one) coefficient to a fixed value that corresponds to a rather low temporal resolution [29], which can deviate from the real value when going to very high temporal resolution. As a result t-values can be overestimated because the order of the respective Student t-distribution is estimated to be too large. The second problem is related to the underlying theory of random Gaussian fields to correct the probability of getting a false positive voxel in the whole volume due to spatial correlations between neighbouring points. The assumption that the spatial correlations between points can be accurately described by a Gaussian distribution [30] is not true anymore, since spatial correlations are now introduced by more complicated point spread functions due to the form of the trajectory. A proper statistical analysis of functional data reconstructed from non-cartesian k-space data with high temporal resolution certainly needs more attention.

For Tikhonov regularization the regularization parameter could be detected automatically using the L-curve method. The method has been chosen due to its robustness and the fact that the encoding matrix is not needed explicitly as in other methods. The task of automatically determining the regularization parameter is more challenging in case of the nonlinear regularization. Methods that work well in the Tikhonov case do not necessarily apply to the nonlinear case. Although there is no apparent reason why the L-curve should be problematic when used in combination with nonlinear cost functions, in our experience the corner of the L-curve can be much less pronounced and the corresponding parameter can yield unsuitable solutions. This problem is exacerbated by a lack of an analytic form for the L-curve, which means that in a practical implementation the corner needs to be estimated from a few precomputed points on the curve.

The most prominent physiological signal components (ECG, respiration) introduce significant additional variance in voxel time courses of functional MRI data. In contrast to conventional EPI experiments with volume coverage, where physiological signal changes appear as pseudo-noise in the signal time course, these signal modulations can be adequately resolved with a temporal resolution of 100 ms. Physiological “noise” correction could therefore be performed by modelling ECG and respiration signal changes in the design matrix. This correction method is feasible only because the temporal resolution achieved here prevents noise from being aliased into lower frequencies. In the future, we will investigate further the possibilities for physiological noise removal when dealing with high temporal sampling rates.
